# Supplementary material for: New Caledonian crows plan for specific future tool use
Source: Proc Biol Sci. 2020 Nov 4;287(1938):20201490. doi: 10.1098/rspb.2020.1490 (PMC7735258; doi:10.1098/rspb.2020.1490)
Supplement: Prior Experience, Additional Methods, Additional Results [file rspb20201490supp1.docx]

**Supplementary Information**

**Prior Experience**

**Tool Use Training:** To train crows to use a stone as a tool, subjects were first trained to drop stones into the dispenser apparatus by placing small pieces of meat underneath a stone, which was placed on the edge to the opening of the dispenser. The pieces of meat gradually decreased in size and once the crow started pushing the stone in without any meat, the stones were gradually placed further away so the crow had to carry the stones to the dispenser. A vertical tube was then placed next to the dispenser apparatus and crows learnt to drop the stone into it. Finally, the crows were trained to drop stones into the platform apparatus in order to get food. We used a similar procedure for the hook-dispenser combination. Crows did not need training to use a stick tool, due to their natural tool behaviors in the wild, but were given habituation and experience with the tube apparatus so they were competent at getting food from it.

**Tool Selection Training:** We presented crows with one of the apparatuses (tube or platform) and the two tools (stick and stone). One of the two presented tools was positioned 20cm to the left of the apparatus, the second one was placed 20cm to the right of the apparatus. Crows received blocks of 10 trials where both the apparatus type, and the position of the tools, was counterbalanced and randomized across trials. Crows had to take the correct tool in order to gain the reward and were trained until they reached a criterion of 17 out of 20 correct trials across two consecutive blocks (binomial choice with alpha set at 0.01). Crows were allowed to pick up the incorrect tool, put it down and then pick up the second tool (i.e. switch tools), as long as they made no contact with the apparatus with the incorrect tool. However, as soon as the incorrect tools was placed in contact with the apparatus the trial was counted as failed. Once crows reached criterion they were tested to a second criterion of 17/20 where tool switches were counted as a failure and interrupted. This procedure was used to teach the crows that their first tool choice had to be correct. (see [1] for details)

**Quality Allocation Training:** To give crows experience of making decisions about high- and low-quality food, they were presented with either a stone or stick apparatus that contained either meat (high value food reward) or apple (low value food reward). A piece of food, opposite in value to the one inside the apparatus, was placed either to the left or the right of the apparatus, and a functional tool was placed on the other side of the apparatus. Crows therefore had to decide whether to use the tool or take the available food, depending on whether meat had been placed inside or outside of the apparatus. The apparatuses, location of the meat and the position of the tool was counterbalanced and randomized across trials. The criterion was 17/20 correct trials across two consecutive blocks in order to proceed to the next step. (see [1] for details)

**Apparatus Functionality Training**: To ensure crows understood the functionality of each tool with each apparatus, a platform and a tube apparatus, both baited with meat, were presented along with one tool. Crows had to take this tool to the correct apparatus in 17/20 trials across two consecutive blocks. The position of apparatuses and the available tool (stone/stick) were randomized and counterbalanced across trials. Once this test was passed, crows were given a second test where both apparatuses and both tools were presented, but only one of the two apparatuses was baited with meat, the other one remained empty. Crows had to take the correct tool to the baited apparatus in 17/20 correct trials across two consecutive blocks. The position of the apparatuses and tools and the position of the meat was randomized and counterbalanced across trials. Finally, a third test was given where both apparatuses and both tools were present, but only one of the apparatuses contained the meat, the other one apple. The crows had to select the correct tool for the apparatus containing meat in 17/20 trials across two consecutive blocks. Again, position of apparatuses, tools and the bait was counterbalanced and randomized across trials. (see [1] for details)

**Mental Representation Training:** Crows were presented with one apparatus (either tube or platform) and the stick and stone tool, which were presented together either 20cm to the left or to the right of the apparatus. Both the location of the tools and the identity of the apparatus was randomized and counterbalanced across trials. In Test 1, a visual barrier was introduced and positioned either on the right or the left of the apparatus. Both tools were placed together behind the barrier and therefore the crow was only able to see the apparatus or the tools at one time. The crow had to choose the correct one of the two tools and take it to the apparatus until it scored 17/20. In Test 2, a second visual barrier was placed on the other side of the apparatus, and the two tools were now split up, with each one put behind one of the two barriers. Now the crows could not see more than one of the three components of the task at the same time (stick tool, stone tool, apparatus). The position of the tools and apparatus was counterbalanced and randomized across trials and crows were tested until they picked the correct tool in 17/20 trials. Test 1 and Test 2 were then repeated but with the barrier replaced with a large wooden four-sided shield that was used in metatool experiments (see below for details).

**Hook Training and Tool Transport Training:** The crows were next given experience dropping the hook tool into the dispenser apparatus until they were reliably able to get food. They were then given experience carrying the stick, the stone and the hook around the aviary. This training began with carrying the tool around a single compartment (e.g. from a branch to the table) and was then increased to carrying between compartments, until crows had carried each tool from the table in one compartment to the apparatus location in another. Training criterion was to transport the correct tool to the apparatus in 7 out of 10 trials. We introduced tool transport training as the wild-caught birds had trouble transporting tools while experimenters moved around the aviary and opened doors. Due to these actions birds often dropped the tool, and transport training was necessary to provide experience with the procedure. This training ensured that if the crows failed, it could be attributed to a lack of planning ability, rather than a lack of comfort transporting tools. This does not affect our conclusions because our test was to see if crows would transport the correct tool for the correct job, rather than spontaneously transport a tool generally, as has been tested for in past studies.

**Five Choice Tool Functionality Training:** Crows were presented with the five choice Perspex tool presentation box that would be used in the study. It contained all three tools (stick, stone and hook) with the tools’ position pseudo-randomised between all five boxes across trials. Crows were also presented with the dispenser apparatus next to the tool presentation box. Once crows chose the hook in 17/20 correct trials they were presented with the platform apparatus until they again reached criterion, at which point they were presented with the tube apparatus. Crows were then presented with trials where the three apparatuses were randomly swapped between trials, again until they achieved a criterion of 17/20.

**Metatool Experience:** We next ran three metatool experiments (see [2] for details) where crows had to use a tool to gain access to another tool, which could then be used to gain food. Each stage of these problems was placed on a different side of the four-sided metatool shield, so requiring crows to mentally represent the location and identity of either tools or apparatuses in order to solve the problem. All crows were given this experience, aside from Mercury and Uranus who did not receive these tests before participating in this experiment.

**Delayed gratification experience**: Birds were given two trial types. In the first a large piece of meat was placed inside the tube apparatus and a stick and a small piece of meat were available in front of the tube. In the second the large piece of meat was replaced with apple. Thus, the optimal decision was to use to tool to get the meat in the first trial type, but to take the small piece of meat, rather than the tool, in the second trial type. Birds had to select optimally in 17/20 trials to reach criterion. (see [3] for details)

**Experimental Follow-up**

After the crows had completed Conditions 3 and 4, we ran an additional follow-up. Here, we repeated Conditions 3 and 4, but 30 seconds into inspection phase, we visibly removed the baited apparatus (so there would be no baited apparatus available in the future (in accession phase) (Conditions 5 & 6). We also changed the food presented at test from apple to meat. We found that all three crows chose the meat in each of the first six trials that they were given (Binomial choice between five choices, p >0.001). These results clearly show that the tools had not become so associated with food that they were preferred over freely available meat.

**2017 Pilot Planning Study**

Below we describe the 2017 pilot data we mentioned in our pre-registration [4]. This experiment was conducted with different birds than those used in our 2018 study.

**Material & Methods**

**Subjects:** In 2017 we tested six wild caught New Caledonian crows (*Corvus moneduloides*) on the island of Grand Terre, New Caledonia. Four crows were adults older than two years (Janis, David, Elvis, and Bob), two were juveniles younger than one year (Freddie, Aretha). Two individuals were identified as females based on the sexual size dimorphism [5] (Janis and Aretha) and the other four were males.

**Apparatus:** The same three apparatuses (tube, platform and dispenser) were used in the 2017 pilot as in the 2018 study but with different individuals.

**Prior Experience:** Before the birds were tested, they participated in a tool functionality study (similar to the tool selection, quality allocation and apparatus functionality training in the pre-experience section of the SI method above). All crows additionally participated in a mental representation study and were given training on tool transportation. These were similar to the mental representation training and tool transportation training described in the pre-experience section of the SI method above. They were also given dispenser functionality training, where the crows were presented with the dispenser and either a hook and stick or hook and stone, until they chose the hook correctly in 10/12 trials. All birds were then given experience with a delayed gratification task, again similar to that described in the pre-experience section of the SI method above. Finally, three birds, Janice, David and Freddie also participated in a metatool experiment similar to the one described above, before being given the planning study.

**Procedure**

**Training:** We used the tube apparatus in the training phase. In Condition 1, birds first observed a tube apparatus baited with 10 pieces of meat for 1 minute in one compartment. After this time, the bird was moved to a second compartment. After 5 minutes, the birds were presented with a forced choice between a stick and a very small piece of meat. After 15 minutes, birds were allowed to go back into compartment 1 in order to interact with the apparatus. If birds had chosen the stick, they were able to retrieve the food. In Condition 2, birds again observed the tube apparatus but this time it was baited with apple, a less preferred food. They were then presented with the same temporal sequence. The optimal decision now was to choose the meat after 5 minutes, because the stick could only be used to get apple from the tube. Birds received multiple repetitions of Conditions 1 and 2 until they chose correctly in 6/6 consecutive trials across both conditions.

**Test:** At test crows were presented with alternating trials where they either observed a platform apparatus, or a dispenser apparatus, for one minute, and then 5 minutes later in the adjacent room were given the choice of a hook, stone or a stick tool. The optimal decision was to choose the stone when they observed the platform apparatus and the hook when they observed the dispenser apparatus. Birds were presented with 6 trials in total. While none of the birds performed above chance due to low trial numbers, one scored 5/6 and two others scored 4/6 (Table S4, Figure S1).

**Table S4.** Correct Choices in Conditions 1-4.

|  |  | Correct | Total | % | Binomial |
| --- | --- | --- | --- | --- | --- |
| C1&2 | Annie | 8 | 10 | 80 | 0.044 |
|  | David | 8 | 9 | 89 | 0.018 |
|  | Freddie | 8 | 9 | 89 | 0.018 |
|  | Janis | 11 | 17 | 65 | 0.094 |
| C3&4 | Annie | 4 | 6 | 67 | 0.234 |
|  | David | 3 | 6 | 50 | 0.313 |
|  | Freddie | 4 | 6 | 67 | 0.234 |
|  | Janis | 5 | 6 | 83 | 0.094 |

**Figure S1: Choices in Training and Experiment.** Selection of the correct (1) and the incorrect choice in the training phase (blue, Conditions 1-2) and the experimental phase (red, Conditions 304).

**Movie S1: Examples of Crows Solving the Future Planning Tasks.** Condition 3 - Trial 1: apparatus inspection, forced choice, tool transportation, and solution of stone platform combination by Neptune. Condition 4 – Trial 1: apparatus inspection, forced choice, tool transportation, and solution of hook dispenser combination by Neptune. Condition 3 - Trial 2: apparatus inspection, forced choice, tool transportation, and solution of stone platform combination by Neptune. Condition 4 – Trial 2: apparatus inspection, forced choice, tool transportation, and solution of hook dispenser combination by Neptune.

Temporary Link until publication:

<https://www.dropbox.com/s/9hsei8z3bb5ia1k/Neptune%2B4%2BTrials%2BC3%2526C4%2BSupplement%2BV10.mp4?dl=0>

Literature:

[1] Miller, R., Gruber, R., Frohnwieser, A., Schiestl, M., Jelbert, S.A., Gray, R.D., Boeckle, M., Taylor, A.H. & Clayton, N.S. 2020 Decision-making flexibility in New Caledonian crows, young children and adult humans in a multi-dimensional tool-use task. *PloS one* **15**, e0219874.

[2] Gruber, R., Schiestl, M., Boeckle, M., Frohnwieser, A., Miller, R., Gray, R.D., Clayton, N.S. & Taylor, A.H. 2019 New Caledonian crows use mental representations to solve metatool problems. *Current Biology* **29**, 686-692. e683.

[3] Miller, R., Frohnwieser, A., Schiestl, M., McCoy, D.E., Gray, R.D., Taylor, A.H. & Clayton, N.S. 2020 Delayed gratification in New Caledonian crows and young children: influence of reward type and visibility. *Animal cognition* **23**, 71-85.

[4] Boeckle, M., Schiestl, M., Frohnwieser, A., Gruber, R., Miller, R., Suddendorf, T., Gray, R.D., Taylor, A.H. & Clayton, N.S. 2018 Flexible Planning. In *The Open Science Forum* (The Open Science Framework.

[5] Kenward, B., Rutz, C., Weir, A.A.S., Chappell, J. & Kacelnik, A. 2004 Morphology and sexual dimorphism of the New Caledonian Crow Corvus moneduloides, with notes on its behaviour and ecology. *Ibis* **146**, 652-660.
